# Assessment of torso deformities using 3D markerless asymmetry analysis and its clinical applications

**DOI:** 10.1186/1748-7161-8-S2-O25

**Published:** 2013-09-18

**Authors:** Amin Komeili, Lindsey Westover, Eric Parent, Marc Moreau, Marwan El-Rich, Samer Adeeb

**Affiliations:** 1 University of Alberta, Alberta, Canada

## Background

The location of the scoliosis curve apex is important in brace prescription and can only be obtained from radiographs[1]. Surface Topography (ST) has poor correlation with curve shape but current ST analyses often rely on markers placed by operators, which can affect measurement accuracy.

## Purpose

This study aimed to meet the following objectives: (1) develop an approach to analyzing markerless torso ST data, (2) propose a ST classification system for torso asymmetry in adolescent idiopathic scoliosis (AIS) patients, and (3) predict the location of the curve apex based on ST.

## Methods

The full torso ST of 90 AIS patients with different curve types were retrieved from our previous study [2]. The mean Cobb angle was 32.5° (range: 8°-69°). The best plane of symmetry that divides the torso into left and right parts was calculated. Deviations between the left and right parts were measured and displayed as deviation colour maps (DCMs). To propose a surface clarification, the DCMs of 46 patients were appraised by three scoliosis professionals. The DCMs were then classified into three main groups and six subgroups by four novice observers. The intra and inter-observer reliability of the classification was assessed using Kappa coefficients. The vertical position of the maximum deviation point above the PSISs was multiplied by a correction factor to estimate the vertical location of the curve apex.

## Results

The mean kappa coefficient for intra-observer reliability was 0.85 (0.68-0.92) indicating good to excellent classification reliability [3]. The inter-observer kappa coefficient was 0.62 and the percentage of agreement was 80%, indicating moderate reliability[3]. For 88 torsos with thoracic curves (subgroups 2, 4 or 5), the location of the point of maximum deviation predicted the location of the curve apex with a ±2.2cm accuracy (range 0.02-5.5cm, R^2^=0.72). For 39 lumbar curves with Cobb angle >20^o^_,_ prediction accuracy was ±1.6cm (0.02-4.2cm; R^2^=0.42).

## Conclusions and discussion

A non-invasive markerless ST quantification of the asymmetry of the torso was developed allowing reliable classification of patients with scoliosis. The new approach can predict the location of the curve apex with errors corresponding to the dimensions of one vertebra.
